# Supplementary material for: Performance of cardiopulmonary exercise testing for the prediction of post-operative complications in non cardiopulmonary surgery: A systematic review
Source: PLoS One. 2020 Feb 3;15(2):e0226480. doi: 10.1371/journal.pone.0226480 (PMC6996804; doi:10.1371/journal.pone.0226480)
Supplement: S1 Appendix — Example given for repeat search—same terms used for initial search, see Methods. (PDF) [file pone.0226480.s001.pdf]

S1 Appendix: Literature Search Strategy (example given for repeat search – same terms used for initial search, see methods)

Database: Ovid MEDLINE(R) and Epub Ahead of Print, In-Process & Other Non-Indexed Citations, Daily, and Versions(R) <1946 to July 24, 2018>

Search Strategy:

----

1 exp postoperative complication/ or postoperative.mp. or exp postoperative period/ (827536)

2 post?operative.mp. (715899)

3 post?surg*.mp. (17054)

4 post?an?esth*.mp. (4123)

5 ((post* or after*) adj3 (surg* or operat* or an?esth*)).mp. [mp=title, abstract, original title, name of substance word, subject heading word, floating sub-heading word, keyword heading word, protocol supplementary concept word, rare disease supplementary concept word, unique identifier, synonyms] (448032)

6 myocardial infarction.mp. or exp heart infarction/ (225744)

7 exp acute heart infarction/ or exp heart infarction/ or MI.mp. (41812)

8 cardiac arrest.mp. or exp heart arrest/ (55523)

9 arrhythmia.mp. or exp heart arrhythmia/ (64504)

10 heart failure.mp. or exp heart failure/ (182275)

11 congestive cardiac failure.mp. or exp congestive heart failure/ (108906)

12 angina.mp. or exp angina pectoris/ (67035)

13 stroke.mp. or exp cerebrovascular accident/ (272135)

14 major adverse cardiac event*.mp. (5433)

15 (MACE or MACCE).mp. [mp=title, abstract, original title, name of substance word, subject heading word, floating sub-heading word, keyword heading word, protocol supplementary concept word, rare disease supplementary concept word, unique identifier, synonyms] (6493)

16 exp death/ or death.mp. (758831)

17 exp mortality/ or mortality.mp. (1112088)

18 ((unplanned or unexpect* or unintentional or unnecessary) adj3 (ITU or ICU or intensive care unit)).mp. [mp=title, abstract, original title, name of substance word, subject heading word, floating sub-heading word, keyword heading word, protocol supplementary concept word, rare disease supplementary concept word, unique identifier, synonyms] (345)

19 exp pneumonia/ or exp aspiration pneumonia/ or exp hospital acquired pneumonia/ or pneumonia.mp. (141779)

20 (respiratory infection or LRTI or lower respiratory tract infection).mp. [mp=title, abstract, original title, name of substance word, subject heading word, floating sub-heading word, keyword heading word, protocol supplementary concept word, rare disease supplementary concept word, unique identifier, synonyms] (11150)

21 respiratory failure.mp. or exp respiratory failure/ (76013)

22 exp hypoxia/ or hypoxia.mp. (142339)
23 hypercarbia.mp. or exp hypercapnia/ (9227)
24 pleural effusion.mp. or exp pleura effusion/ (27496)
25 pneumothorax.mp. or exp pneumothorax/ (25299)
26 bronchospasm.mp. or exp bronchospasm/ (7245)
27 atelectasis.mp. or exp atelectasis/ (10708)
28 (aspiration adj pneumonitis).mp. [mp=title, abstract, original title, name of substance word, subject heading word, floating sub-heading word, keyword heading word, protocol supplementary concept word, rare disease supplementary concept word, unique identifier, synonyms] (460)
29 cardio?pulmonary exercise test.mp. or exp cardiopulmonary exercise test/ (59968)
30 CPET.mp. (1036)
31 CPEX.mp. (17)
32 or/1-5 (1087570)
33 or/6-28 (2556975)
34 or/29-31 (60238)
35 and/32-34 (1225)
36 limit 35 to yr="2017 -Current" (38)
