# Supplementary material for: Performance of cardiopulmonary exercise testing for the prediction of post-operative complications in non cardiopulmonary surgery: A systematic review
Source: PLoS One. 2020 Feb 3;15(2):e0226480. doi: 10.1371/journal.pone.0226480 (PMC6996804; doi:10.1371/journal.pone.0226480)
Supplement: S3 Appendix — (PDF) [file pone.0226480.s003.pdf]

Systematic review

To edit the record click *Start an update* below. This will create a new version of the record - the existing version will remain unchanged.

1. * Review title.

Give the working title of the review, for example the one used for obtaining funding. Ideally the title should state succinctly the interventions or exposures being reviewed and the associated health or social problems. Where appropriate, the title should use the PI(E)COS structure to contain information on the Participants, Intervention (or Exposure) and Comparison groups, the Outcomes to be measured and Study designs to be included.

Metrics of classifier performance for Cardiopulmonary Exercise Testing in the identification of patients at risk of peri-operative complications: a systematic review

2. Original language title.

For reviews in languages other than English, this field should be used to enter the title in the language of the review. This will be displayed together with the English language title.

3. * Anticipated or actual start date.

Give the date when the systematic review commenced, or is expected to commence.

20/03/2017

4. * Anticipated completion date.

Give the date by which the review is expected to be completed.

30/11/2018

5. * Stage of review at time of this submission.

Indicate the stage of progress of the review by ticking the relevant Started and Completed boxes. Additional information may be added in the free text box provided.

Please note: Reviews that have progressed beyond the point of completing data extraction at the time of initial registration are not eligible for inclusion in PROSPERO. Should evidence of incorrect status and/or completion date being supplied at the time of submission come to light, the content of the PROSPERO record will be removed leaving only the title and named contact details and a statement that inaccuracies in the stage of the review date had been identified.

This field should be updated when any amendments are made to a published record and on completion and publication of the review. If this field was pre-populated from the initial screening questions then you are not able to edit it until the record is published.

The review has not yet started: No

Review stage	Started	Completed
Preliminary searches	Yes	Yes
Piloting of the study selection process	Yes	Yes
Formal screening of search results against eligibility criteria	Yes	Yes
Data extraction	Yes	No
Risk of bias (quality) assessment	No	No

Review stage	Started	Completed
Data analysis	No	No

Provide any other relevant information about the stage of the review here (e.g. Funded proposal, protocol not yet finalised).

6. * Named contact.

The named contact acts as the guarantor for the accuracy of the information presented in the register record.

Daniel Stubbs

Email salutation (e.g. "Dr Smith" or "Joanne") for correspondence:
Dr Stubbs

7. * Named contact email.

Give the electronic mail address of the named contact.

djs225@cam.ac.uk

8. Named contact address

PLEASE NOTE this information will be published in the PROSPERO record so please do not enter private information

Give the full postal address for the named contact.

University Division of Anaesthesia, Addenbrooke's Hospital, Hills Road, Cambridge, Cb2 0QQ

9. Named contact phone number.

Give the telephone number for the named contact, including international dialling code.

+44 (0)1223 245151

10. * Organisational affiliation of the review.

Full title of the organisational affiliations for this review and website address if available. This field may be completed as 'None' if the review is not affiliated to any organisation.

University Division of Anaesthesia

Organisation web address:

11. * Review team members and their organisational affiliations.

Give the title, first name, last name and the organisational affiliations of each member of the review team. Affiliation refers to groups or organisations to which review team members belong.

Dr Daniel Stubbs. University Division of Anaesthesia
Dr Lisa Grimes. University Division of Anaesthesia
Dr Ari Ercole. University Division of Anaesthesia

12. * Funding sources/sponsors.

Give details of the individuals, organizations, groups or other legal entities who take responsibility for initiating, managing, sponsoring and/or financing the review. Include any unique identification numbers assigned to the review by the individuals or bodies listed.

None.

13. * Conflicts of interest.

List any conditions that could lead to actual or perceived undue influence on judgements concerning the main topic investigated in the review.

None

14. Collaborators.

Give the name and affiliation of any individuals or organisations who are working on the review but who are not listed as review team members.

15. * Review question.

State the question(s) to be addressed by the review, clearly and precisely. Review questions may be specific or broad. It may be appropriate to break very broad questions down into a series of related more specific questions. Questions may be framed or refined using PI(E)COS where relevant.

Is it possible to generate estimates of key classifier metrics (such as sensitivity and specificity) describing cardiopulmonary exercise testing's (CPET) ability to prognosticate adverse outcome in patients undergoing major non cardiopulmonary surgery?

16. * Searches.

State the sources that will be searched. Give the search dates, and any restrictions (e.g. language or publication period). Do NOT enter the full search strategy (it may be provided as a link or attachment.)

Databases used: EMBASE, MEDLINE

Search strategy: Identification of time period of interest (e.g. post-operative, post-surgical, post-anaesthetic) + complications of interest (cardiopulmonary, death, unplanned ICU) + cardiopulmonary testing, CPET, CPEX

Where appropriate we will search titles, abstracts and use MeSH terms. There will be no date restriction. We will also review references from included articles to determine their suitability for inclusion

At the completion of data extraction we will re-run the search and two authors will screen newly identified articles to ensure that there are no other studies which need to be included

17. URL to search strategy.

Give a link to a published pdf/word document detailing either the search strategy or an example of a search strategy for a specific database if available (including the keywords that will be used in the search strategies), or upload your search strategy.

Do NOT provide links to your search results.

Do not make this file publicly available until the review is complete

18. * Condition or domain being studied.

Give a short description of the disease, condition or healthcare domain being studied. This could include health and wellbeing outcomes.

Prognostication of perioperative complications (cardiopulmonary, death, unplanned ICU admission).

19. * Participants/population.

Give summary criteria for the participants or populations being studied by the review. The preferred format includes details of both inclusion and exclusion criteria.

All adult patients who undergo non cardiopulmonary surgery having undergone cardiopulmonary exercise testing.

20. * Intervention(s), exposure(s).

Give full and clear descriptions or definitions of the nature of the interventions or the exposures to be reviewed.

Patients who have undergone cardiopulmonary exercise testing for the calculation of physiological variables such as (but not limited to) Anaerobic Threshold (AT), ventilatory equivalents for oxygen/carbon dioxide, and peak oxygen uptake.

21. * Comparator(s)/control.

Where relevant, give details of the alternatives against which the main subject/topic of the review will be compared (e.g. another intervention or a non-exposed control group). The preferred format includes details of both inclusion and exclusion criteria.

If present we will generate classifier metrics for other prognostic methods present in the same studies (e.g. american society of anesthesiologists (ASA) score, timed walk tests).

22. * Types of study to be included.

Give details of the types of study (study designs) eligible for inclusion in the review. If there are no restrictions on the types of study design eligible for inclusion, or certain study types are excluded, this should be stated. The preferred format includes details of both inclusion and exclusion criteria.

Any primary research study using variables derived from CPET testing to identify patients at risk of cardiopulmonary complications (as defined by the ESICM), death, or unplanned ICU admission. This could include retrospective case series, cohort studies, or prospective randomised studies.

Studies must have been published in an article (we will exclude those published only as a conference abstract) and we will exclude correspondence, review, editorial and opinion pieces.

23. Context.

Give summary details of the setting and other relevant characteristics which help define the inclusion or exclusion criteria.

24. * Main outcome(s).

Give the pre-specified main (most important) outcomes of the review, including details of how the outcome is defined and measured and when these measurement are made, if these are part of the review inclusion criteria.

Cardiopulmonary complications as defined by the ESICM (will be included even if included in a pooled outcome e.g. cardiac complications alone, respiratory complications alone, pooled cardiorespiratory). Exclude if composite with death.

Timing and effect measures

In-hospital

25. * Additional outcome(s).

List the pre-specified additional outcomes of the review, with a similar level of detail to that required for main outcomes. Where there are no additional outcomes please state 'None' or 'Not applicable' as appropriate to the review

In-hospital and 30 Day mortality, Unplanned ICU admission.

Timing and effect measures

26. * Data extraction (selection and coding).

Describe how studies will be selected for inclusion. State what data will be extracted or obtained. State how this will be done and recorded.

We will extract any reported sensitivities and specificities for one of our chosen outcomes with the corresponding CPET variable and cut-off.

We will also record any AUC (area under the receiver operator curve) values for CPET and other prognostic variables reported in the paper.

If such analysis was not reported in the paper we will record whether the authors found a significant association between their chosen outcome and CPET derived variable as well as the statistical test used.

We will also aim to extract data to generate our own 2x2 classifier tables (confusion matrices). In this process:

-A true positive will be defined as a patient who had a CPET value underneath any specified cut-off in the paper and suffered the outcome in question. (e.g. Anaerobic Threshold < 12 AND died)
- A false positive will be defined as a patient who had a CPET value underneath any specified cut off in the paper and did not suffer the outcome in question (e.g. Anaerobic Threshold < 12 AND lived)
-A true negative' will be defined as a patient whose CPET variables were above any specified cut-off and did not suffer the specified adverse outcome. (e.g. Anaerobic Threshold > 12 AND lived)
- A false negative will be defined as a patient who had a CPET value above the specified cut off in the paper and DID suffer the outcome in question (e.g. Anaerobic Threshold > 12 AND died)

As a secondary aim we will undertake the above process for other examined prognostic variables (e.g. ASA score) within each paper if possible.

All data extraction will be performed independently by DS and LG.
If possible, we will undertake a meta-analysis of all identified data including the generation of summary receiver operator curves (SROC) if enough comparable data points are available.

27. * Risk of bias (quality) assessment.

Describe the method of assessing risk of bias or quality assessment. State which characteristics of the studies will be assessed and any formal risk of bias tools that will be used.

QUIPS for all studies.
CHARMs checklist for those reporting a multivariable predictive model for an outcome of interest.

28. * Strategy for data synthesis.

Provide details of the planned synthesis including a rationale for the methods selected. This **must not be generic text** but should be **specific to your review** and describe how the proposed analysis will be applied to your data.

If possible (depending on data homogeny) we will perform a quantitative analysis with pooled estimates of classifier metrics (including sensitivity, specificity, negative predictive value) for CPET broken down by surgical specialty. SImilarly we will aim to generate summurary receiver operator curves (SROC). If this is not possible due to heterogeneity in the data then we will perform a narrative discussion of individual classifier metrics generated by our data extraction process.

29. * Analysis of subgroups or subsets.

State any planned investigation of 'subgroups'. Be clear and specific about which type of study or participant will be included in each group or covariate investigated. State the planned analytic approach.

Data for each outcome will be presented individually (death, unplanned ICU, cardiopulmonary complications).
To minimise heterogeneity we will aim to generate aggregate values of classifier metrics for individual surgical specialties (e.g. vascular, colorectal, hepatobiliary). Similarly we will present data for individual CPET parameters used as predictive tools in the studies identified (e.g. peak VO2, anaerobic threshold). We will identify for each study whether it was prospective/retrospective and whether clinicians were blinded to CPET values during the study.

30. * Type and method of review.

Select the type of review and the review method from the lists below. Select the health area(s) of interest for your review.

Type of review

Cost effectiveness	No
Diagnostic	No
Epidemiologic	No
Individual patient data (IPD) meta-analysis	No
Intervention	No
Meta-analysis	No
Methodology	No
Narrative synthesis	No

Network meta-analysis	No
Pre-clinical	No
Prevention	No
Prognostic	Yes
Prospective meta-analysis (PMA)	No
Review of reviews	No
Service delivery	No
Synthesis of qualitative studies	No
Systematic review	Yes
Other	No

Health area of the review

Alcohol/substance misuse/abuse	No
Blood and immune system	No
Cancer	No
Cardiovascular	No
Care of the elderly	No
Child health	No
Complementary therapies	No
Crime and justice	No
Dental	No
Digestive system	No
Ear, nose and throat	No
Education	No
Endocrine and metabolic disorders	No
Eye disorders	No
General interest	No
Genetics	No
Health inequalities/health equity	No
Infections and infestations	No

International development	No
Mental health and behavioural conditions	No
Musculoskeletal	No
Neurological	No
Nursing	No
Obstetrics and gynaecology	No
Oral health	No
Palliative care	No
Perioperative care	Yes
Physiotherapy	No
Pregnancy and childbirth	No
Public health (including social determinants of health)	No
Rehabilitation	No
Respiratory disorders	No
Service delivery	No
Skin disorders	No
Social care	No
Surgery	No
Tropical Medicine	No
Urological	No
Wounds, injuries and accidents	No
Violence and abuse	No

31. Language.

Select each language individually to add it to the list below, use the bin icon to remove any added in error.

There is not an English language summary

32. Country.

Select the country in which the review is being carried out from the drop down list. For multi-national collaborations select all the countries involved.

England

33. Other registration details.

Give the name of any organisation where the systematic review title or protocol is registered (such as with The Campbell Collaboration, or The Joanna Briggs Institute) together with any unique identification number assigned. (N.B. Registration details for Cochrane protocols will be automatically entered). If extracted data will be stored and made available through a repository such as the Systematic Review Data Repository (SRDR), details and a link should be included here. If none, leave blank.

34. Reference and/or URL for published protocol.

Give the citation and link for the published protocol, if there is one

No I do not make this file publicly available until the review is complete

35. Dissemination plans.

Give brief details of plans for communicating essential messages from the review to the appropriate audiences.

Do you intend to publish the review on completion?

Yes

36. Keywords.

Give words or phrases that best describe the review. Separate keywords with a semicolon or new line. Keywords will help users find the review in the Register (the words do not appear in the public record but are included in searches). Be as specific and precise as possible. Avoid acronyms and abbreviations unless these are in wide use.

37. Details of any existing review of the same topic by the same authors.

Give details of earlier versions of the systematic review if an update of an existing review is being registered, including full bibliographic reference if possible.

38. * Current review status.

Review status should be updated when the review is completed and when it is published. For newregistrations the review must be Ongoing.

Review_Ongoing

39. Any additional information.

Provide any other information the review team feel is relevant to the registration of the review.

40. Details of final report/publication(s).

This field should be left empty until details of the completed review are available.
