# Supplementary figures and images for: Performance of cardiopulmonary exercise testing for the prediction of post-operative complications in non cardiopulmonary surgery: A systematic review
Source: PLoS One. 2020 Feb 3;15(2):e0226480. doi: 10.1371/journal.pone.0226480 (PMC6996804; doi:10.1371/journal.pone.0226480)

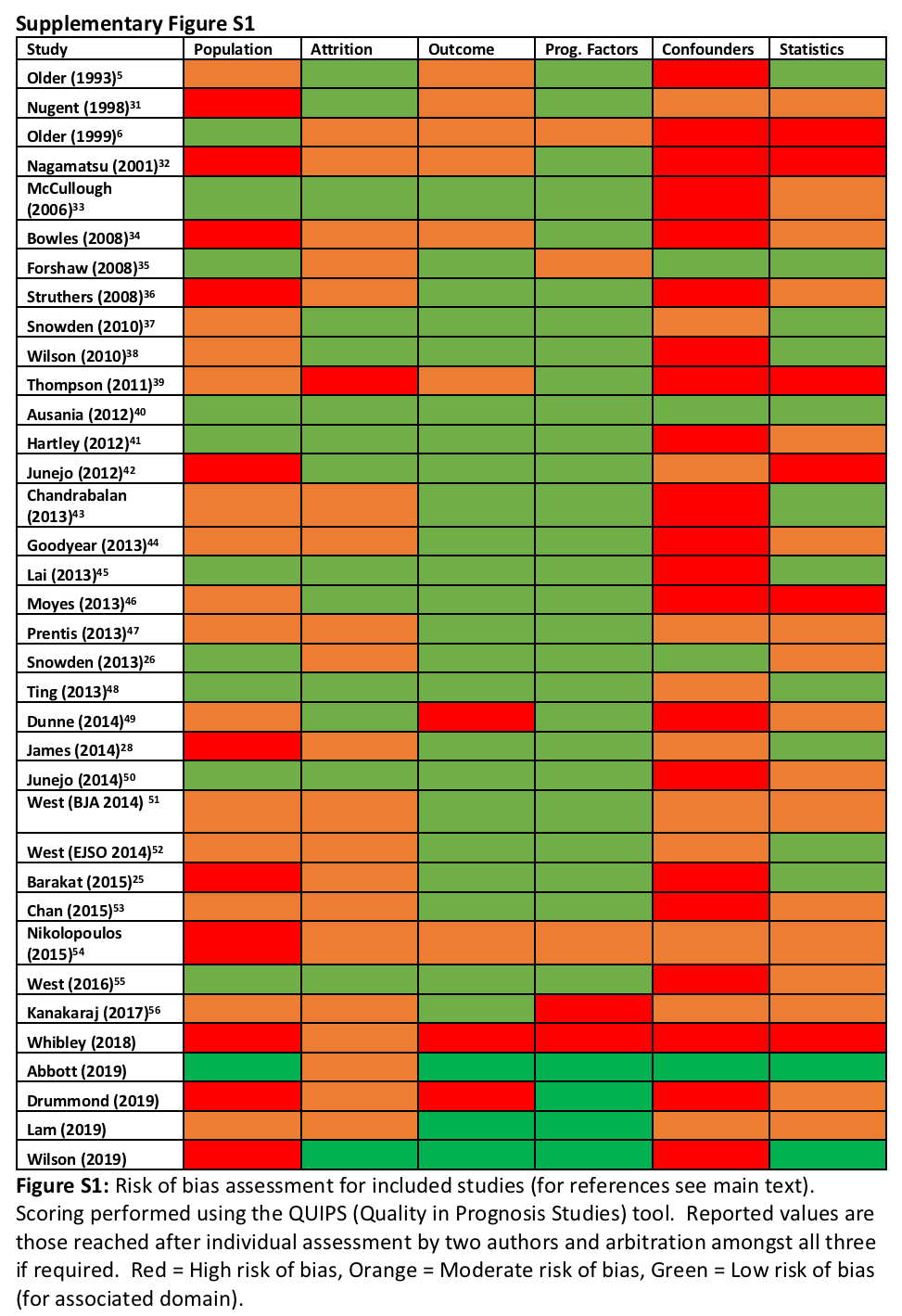

Supplement: S1 Fig — Scoring performed using the QUIPS (Quality in Prognosis Studies) tool. Reported values are those reached after individual assessment by two authors and arbitration amongst all three. Red = High risk of bias, Orange = Moderate risk of bias, Green = Low risk of bias (for associated domain). (TIF) [file pone.0226480.s004.tif]

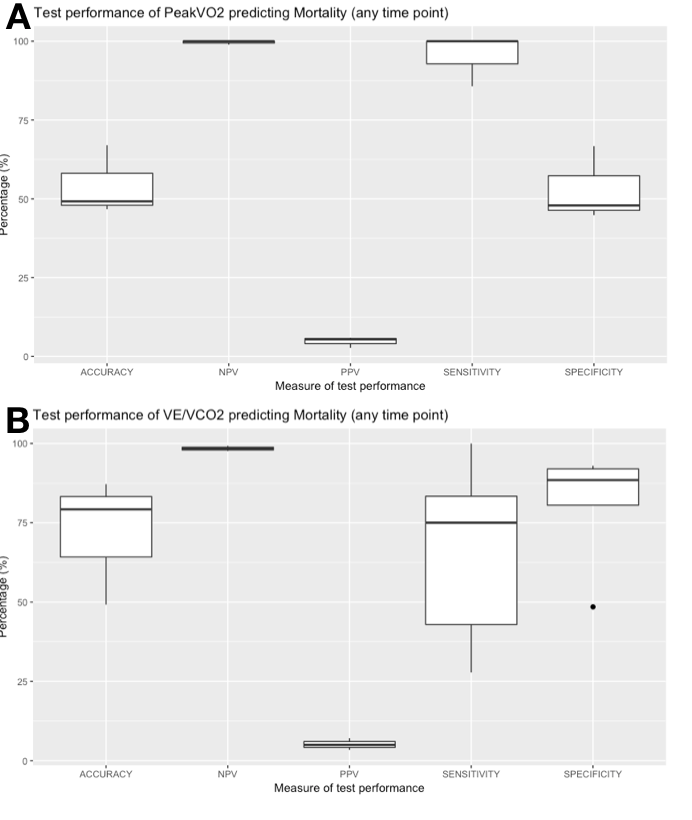

Supplement: S2 Fig — Source data can be seen in table of calculated confusion matrix metric (S1 File). (TIF) [file pone.0226480.s005.tif]
