# Supplementary material for: Performance of cardiopulmonary exercise testing for the prediction of post-operative complications in non cardiopulmonary surgery: A systematic review
Source: PLoS One. 2020 Feb 3;15(2):e0226480. doi: 10.1371/journal.pone.0226480 (PMC6996804; doi:10.1371/journal.pone.0226480)
Supplement: S1 Table — Brackets indicate published 95% confidence interval. Asterisk indicates that published confidence interval crosses 1 (likely calculated as a normal appproximation). (PDF) [file pone.0226480.s007.pdf]

Supplementary Table S1

Study	Outcome	CPET Variable	AUC
**Forshaw (2008)**	Cardiorespiratory	VO2 peak	0.63 [0.5-0.76]
**Junejo (2012)**	Mortality(30D)	AT	0.86
	Mortality (hospital)	AT	0.83
	Cardiorespiratory	VE/VCO2	0.65[0.53-0.77]
**Moyes (2013)**	Cardiorespiratory	AT	0.60[0.48-0.72]
	Cardiorespiratory	VO2 peak	0.62[0.50-0.74]
**Snowden (2013)**	Mortality (hospital)	AT	0.75[0.65-0.85]
**Ting (2013)**	Unplanned ICU	Model(AT+BMI+Desensitisation)	0..93
**James (2014)**	Cardiovascular	AT	0.83[0.69-0.96]
	Cardiovascular	VO2 peak	0.81[0.68-0.93]
**Junejo (2014)**	Mortality(30D)	VE/VCO2	0.95[0.89-1.02]*
	Mortality(Hospital)	VE/VCO2	0.84[0.63-1.07]*
**Nikolopoulos (2015)**	Cardiorespiratory	AT	0.66 [0.35-0.97]
**Kanakaraj (2017)**	Cardiovascular	Peak VO2	0.54[0.27-0.81]
	Cardiovascular	AT	0.57[0.30-0.85]
	Cardiovascular	VE/VCO2	0.73[0.41-1]

Table S1: Published values of area under the receiver operator curve (AUC) from studies. Brackets indicate published 95\% confidence intervals. Asterisk indicates that published confidence interval crosses 1 (likely calculated as a normal approximation)
