# Supplementary material for: Performance of cardiopulmonary exercise testing for the prediction of post-operative complications in non cardiopulmonary surgery: A systematic review
Source: PLoS One. 2020 Feb 3;15(2):e0226480. doi: 10.1371/journal.pone.0226480 (PMC6996804; doi:10.1371/journal.pone.0226480)
Supplement: S2 Table — MET = Metabolic equivalents, BNP = B type natriuretic peptide, NT-proBNP = N-terminal pro b-type natriuretic peptide, eGFR = estimated glomerular filtration rate, CRP = C Reactive Protein, ASA = American Society of Anesthesiologists score, RF = Risk Factors, IHD = Ischaemic Heart Disease, AT = Anaerobic Threshold, RCRI = Revised Cardiac Risk Index. POSSUM = Physiological and Operative Severity Score for the Enumeration of Morbidity and Mortality. AUC = Area under the receiver operator curve, OR = Odds ratio, * calculations exclude 14 high risk patients who didn’t proceed to surgery based ‘partly’ on CPET findings. (PDF) [file pone.0226480.s008.pdf]

Supplementary Table S2

Study	Outcome	Predictor	Finding
**Ausania (2012)**	Mortality	MET Score > 7	Sensitivity 83% Specificity 61%
**Junejo (2012)**	30D Mortality	High v Low risk groups	Sensitivity 100% Specificity 39.2%*
**Junejo (2012)**	In-hosp mortality	High v Low risk groups	Sensitivity 87.5% Specificity 39.6%*
**Junejo (2014)**	30D Mortality	High v Low risk groups)	Sensitivity 100% Specificity 41.3%
**Junejo (2014)**	In-hosp mortality	High v Low risk groups	Sensitivity 100% Specificity 43%
**James (2014)**	MACE	eGFR	AUC:0.72[0.54-0.90]
**James (2014)**	MACE	BNP	AUC:0.75[0.59-0.92]
**James (2014)**	MACE	CRP	AUC:0.56[0.36-0.76]
**James (2014)**	MACE	ASA	AUC:0.68[0.49-0.87]
**James (2014)**	MACE	RCRI	AUC:0.68[0.49-0.86]
**Kanakaraj (2017)**	MACE	NT-proBNP	AUC 0.86[0.73-0.98]
**Bowles (2008)**	Mortality	POSSUM 30	OR:12.6[1.59-100.2]
**Bowles (2008)**	Mortality	ASA 3 or more	OR:11.33[1.43-89.87]
**Bowles (2008)**	Mortality	2 or more comorbidities	OR:6.2[1.9-20.32]
**Wilson (2010)**	Mortality	> 1 Cardiac risk factor	Sensitivity 44.4% Specificity 68.3%
**Wilson (2010)**	Mortality	> 1 Cardiac risk factor+AT <11	Sensitivity 87.5% Specificity 32.7%
**Wilson (2010)**	Mortality	No RF+AT<11	Sensitivity 90.0% Specificity 53.4%
**Wilson (2010)**	Mortality	IHD	Sensitivity 38.9% Specificity 83.6%
**Wilson (2010)**	Mortality	IHD + AT<11	Sensitivity 85.7% Specificity 25.7%

Table S4: Data pertaining to non CPET based predictors explored in included studies. MET = Metabolic equivalents, BNP = B type natriuretic peptide, NT-proBNP = N-terminal pro b-type natriuretic peptide, eGFR = estimated glomerular filtration rate, CRP = C Reactive Protein, ASA = American Society of Anesthesiologists score, RF = Risk Factors, IHD = Ischaemic Heart Disease, AT = Anaerobic Threshold, RCRI = Revised Cardiac Risk Index. POSSUM = Physiological and Operative Severity Score for the Enumeration of Morbidity and Mortality. AUC = Area under the receiver operator curve, OR = Odds ratio, * calculations exclude 14 high risk patients who didn't proceed to surgery based 'partly' on CPET findings and calculated from data in Table 1 in the original paper.
